# M860, a Monoclonal Antibody against Human Lactoferrin, Enhances Tumoricidal Activity of Low Dosage Lactoferrin via Granzyme B Induction

**DOI:** 10.3390/molecules24203640

**Published:** 2019-10-09

**Authors:** Ya Li, Jie Li, Zheng Gong, Xiao-Hua Pan, Zi-Han Ma, Shu-Yan Ma, Hong-Min Wang, Hong-Liang Dong, Fang-Yuan Gong, Xiao-Ming Gao

**Affiliations:** 1 The Institute of Biology and Medical Sciences, Soochow University, Suzhou 215123, China; 2 Key Laboratory of Systemic Biomedicine of Suzhou, Suzhou 215000, China

**Keywords:** Lactoferrin Immune-Complex, Granzyme B, Monocytes, tumoricidal activity

## Abstract

Lactoferrin (LF) is a soluble glycoprotein of the transferring family found in most biological fluids, functioning as a major first line defense molecule against infection in mammals. It also shows certain anti-tumor activity, but its clinical application in tumor therapy is limited because high dosage is required. In this study, we demonstrate that M860, a monoclonal antibody against human LF (hLF), could significantly increase the anti-tumor potential of low dosage hLF by forming LF-containing immune complex (IC). Human monocytes primed with LF-IC, but not hLF or M860 alone, or control ICs, showed strong tumoricidal activity on leukemia cell lines Jurkat and Raji through induction of secreted Granzyme B (GzB). LF-IC is able to colligate membrane-bound CD14 (a TLR4 co-receptor) and FcγRIIa (a low affinity activating Fcγ receptor) on the surface of human monocytes, thereby triggering the Syk-PI3K-AKT-mTOR pathway leading to GzB production. Our work identifies a novel pathway for LF-mediated tumoricidal activity and may extend the clinical application of LF in tumor therapy.

## 1. Introduction

Lactoferrin (LF), an 80-kDa glycoprotein with iron-binding ability, was discovered in mammary secretions and also in the second granules of neutrophils, and could be extracellularly released under conditions of stress or inflammation [1]. It functions as a major defense molecule against infections, and also exhibits certain anti-tumor potential both in vitro and in vivo [2,3]. It has been reported that down-regulation of LF gene in cells leads to an increase of malignant tumors [4,5]. LF and its derivatives induced apoptosis of tumor cells through both extrinsic and intrinsic pathways [3,6]. LF could also inhibit phosphorylation of AKT thus inducing cell cycle arrest of tumor cells [7,8]. In addition to the direct function of LF on tumor cells, LF could also affect immune cells to evade cancers. Bovine LF could significantly improve the anti-tumor activity of NK cells and macrophages [9,10]. As a multifunctional protein, LF is widely used for various clinical conditions. For instance, it is utilized for treating stomach and intestinal ulcers [11,12]. It is also applied as an antioxidant to protect against infections or tissue damage associated with aging [13,14,15]. However, clinical application of LF in tumor therapy is limited because of fast clearance of LF in vivo and high dosage requirement for effective therapy [16,17].

M860, an anti-human LF (hLF) monoclonal antibody produced in this laboratory, can form immune-complex (IC) with hLF [18]. We have previously demonstrated that LF-IC (M860+hLF) was very potent in inducing pro-inflammatory cytokines including TNFα by human monocytes/macrophages [19]. Tumor-associated macrophages (TAM) are mostly of M2 phenotype and capable of promoting tumor growth by secreting immune-suppressive factors [20]. Interestingly, LF-IC could drive M2 to M1 conversion, implying a possible anti-tumor effect of this unique IC [21].

Monocytes have recently emerged as important regulators of cancer development and progression [22,23], through phagocytosis or direct killing of tumor cells, secretion of tumoricidal mediators and differentiation into tyrosine-based activation motifs (TAMs) [24,25]. When exposed to IFN-γ or IFN-α, monocytes could produce soluble protein TRAIL, thereby inducing cell death in cancer cells [26]. On the other hand, however, monocytes could also secrete factors that promote tumor cell invasion [27]. Therefore, monocytes exhibit Janus effects on tumorigenesis. Setting monocytes in an anti-tumor mode represents a useful strategy for treatment against malignancies in vivo. In the present study, we investigate whether and how low combination of M860 and dose hLF could render human monocytes strong tumoricidal activity.

## 2. Results

### 2.1. Human Monocytes Acquire Potent Tumoricidal Activity Following LF-IC Stimulation

**Figure 1:**
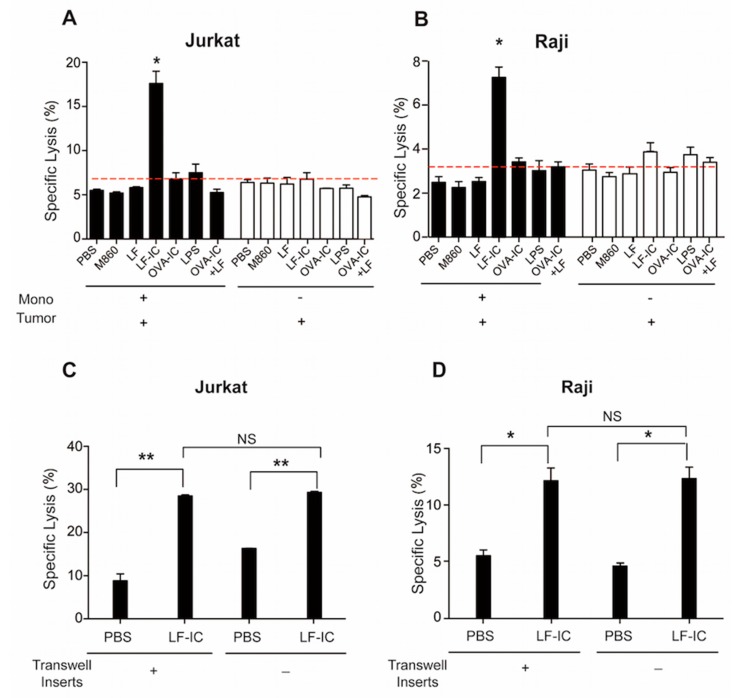
Human monocytes acquire tumoricidal activity after LF-IC stimulation. (**A**,**B**) Freshly purified human monocytes were primed with 30 μg/mL LF, M860, LF-IC (M860+LF), OVA-IC (OVA+M562), OVA-IC +LF or 10 ng/mL LPS for 24 h. CFSE labeled Jurkat (**A**) or Raji (**B**) were then added and incubated for 18 h, with Jurkat or Raji cells treated with LF, M860, LF-IC, OVA-IC as negative controls. (**C**,**D**) Freshly purified human monocytes were primed with 30 μg/mL LF-IC. CFSE labeled Jurkat or Raji cells were seeded directly to the culture or were placed in transwell inserts. Cells were acquired and analyzed by Flow Cytometry with Propidium Iodide staining post co-culture for 18 h. Specific Lysis were expressed by the percentage of CFSE^+^PI^+^ cell counts in CFSE^+^ cell counts. * *p* < 0.05, ** *p* < 0.01.

Based upon our previous findings that LF-IC induces a large amount of TNF-α production by human monocytes via co-ligation of CD14/TLR4 and FcγRIIa (CD32a) [19], we hypothesized that LF-IC might be able to render human monocytes tumoricidal activity due to its Cd14/TLR4-FcγRIIa co-ligation property. Freshly purified human blood monocytes were primed for 24 h with either LF-IC (M860+LF), or hLF, or M860, or complex between chicken ovalbumin (OVA) and OVA-specific mouse mAb M562 (OVA-IC), followed by evaluation for ability to kill leukemia line cells of Jurkat and Raji in vitro. Figs. 1A & 1B show that human monocytes acquired potent tumoricidal activity after treatment with LF-IC, but not with hLF or M860 alone, or OVA-IC, or OVA-IC+hLF. The mAb M562 in OVA-IC is also of mouse IgG1 isotype [19], which is expected to bind FcγRs on monocyte surface in the same manner as M860, yet OVA-IC was unable to activate human monocytes. This is likely due to the lack of CD14/TLR-binding ability of OVA-IC. Importantly, a mixture of hLF and OVA-IC, which was expected to activate both CD14/TLR4 as well as FcγRs, did not show positive effects, indicating that the CD14/TLR4-FcγRIIa co-ligation property of LF-IC is of critical importance for its anti-tumor potential.

Next we addressed the question if the tumoricidal effect of LF-IC-stimulated monocytes was mediated by soluble factors using transwell assays. Primary human monocytes and CFSE-labeled Jurkat, or Raji, cells were cultured in lower and upper (insert) wells, respectively, of transwell chambers for 18 h in the presence of LF-IC. This system prevents direct cell-cell contact but allows tumor cells access to soluble factors secreted by responder monocytes in the opposite wells of the same chambers. Results presented in Figure 1C,D indicate that supernatant from the LF-IC-stimulated human monocytes was sufficient to cause tumor cell death, indicating that the tumoricidal function of LF-IC-primed monocytes was mediated via soluble factors and independent of direct cell-cell contact.

### 2.2. The Tumoricidal Function of LF-IC-Primed Human Monocytes Is Independent of TNFα Production

**Figure 2:**
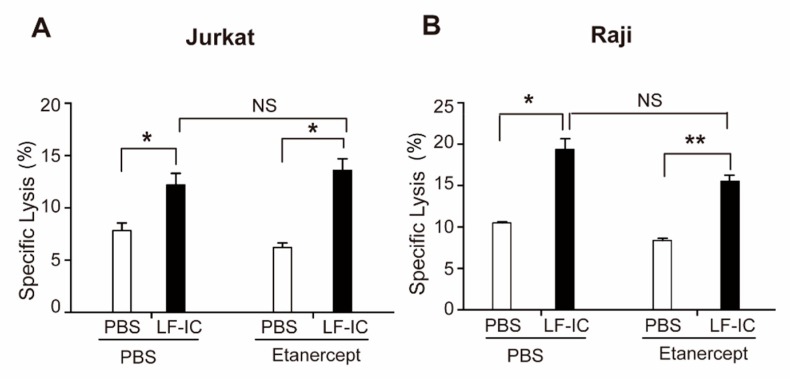
The tumoricidal function of LF-IC-primed human monocytes is independent of TNFα secretion. LF-IC primed monocytes were treated by 100 μg/mL Etanercept or left untreated for 24 h. CFSE labeled Jurkat (**A**) or Raji (**B**) were then added and co-cultured with primed monocytes for 18 h. Cells were acquired and analyzed by FACS with PI staining. Results were expressed by the percentage of CFSE^+^PI^+^ cell counts in CFSE^+^ cell counts. * *p* < 0.05, ** *p* < 0.01.

TNFα is known to trigger apoptosis in tumor cells through blocking NF-κB signaling [28,29], and thus considered a possible mediator of the tumoricidal effect of LF-IC-primed monocytes. However, Etanercept, a blocking mAb against TNFα, did not affect the tumoricidal activity of LF-IC primed monocytes (Figure 2), arguing for the presence of soluble factors other than TNFα responsible for the killing of the tumor cells in these experiments.

### 2.3. Granzyme B Is a Key Mediator of the Tumoricidal Function of LF-IC-Primed Monocytes

**Figure 3:**
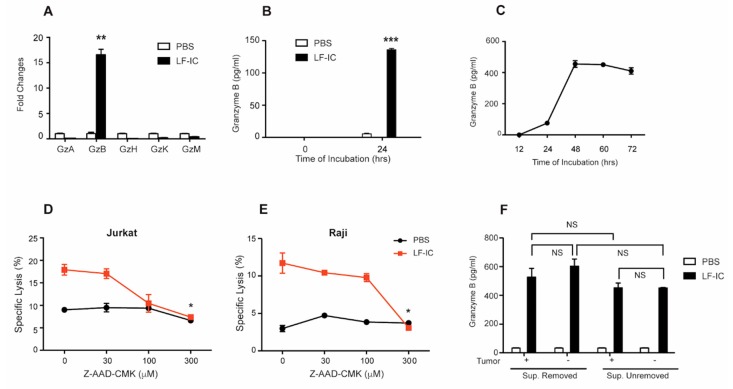
The tumoricidal function of LF-IC-primed human monocytes is dependent on Granzyme B production. Freshly purified human monocytes were primed with 30 μg/mL M860-IC for 24 h. RNA were extracted and expression of Granzyme family were tested by q-PCR (**A**). GzB in Supernatants was determined by ELISA (**B**). (**C**) Freshly purified human monocytes were primed with 30 μg/mL LF-IC for 12, 24, 48, 60, 72 h. GzB in Supernatants was determined by ELISA. (**D**,**E**) Freshly purified human monocytes were primed with 30 μg/mL LF-IC or PBS for 24 h followed by a further incubation with Jurkat (**D**) or Raji (**E**) in the presence of concentrations of GzB inhibitors (Z-AAD-CMK) for 18 h. Cells were acquired and analyzed by FACS with PI staining. Results were expressed by the percentage of CFSE^+^PI^+^ cell counts in CFSE^+^ cell counts. (**F**) Freshly purified human monocytes were primed with 30 μg/mL LF-IC for 24 h. Supernatant were removed and tumor cells were added followed by further incubation for 24 h. In a parallel group, tumor cells were added without supernatant removement. GzB in supernatants was determined by ELISA. * *p* < 0.05, ** *p* < 0.01, *** *p* < 0.001.

Based on our analysis on the differentially expressed genes (DEGs) of RNA-seq data from LF-IC- and OVA-IC-primed human monocytes [30], Granzyme B (GzB), a potent cytotoxic protein produced by myeloid cells [31,32], was amongst the most significantly up-regulated genes in LF-IC-primed cells, which was confirmed by q-PCR (Figure 3A) and ELISA (Figure 3B) results. Furthermore, concentration of GzB in supernatant of LF-IC-stimulated monocyte cultures reached plateau by 48 h (Figure 3C). It has also been reported that Z-AAD-CMK could bind to GzB and irreversibly inhibit its cytotoxic activity. Consistently, the tumoricidal activity of LF-IC-primed human monocytes was dose-dependently inhibited by Z-AAD-CMK (Figs. 3D& 3E). Since tumor cells are known to polarize monocytes through secreted soluble factors or exosomes [20], Jurkat or Raji cells might also be able to induce GzB expression by human monocytes. However, concentration of GzB in the supernatant of LF-IC primed monocyte cultures was unaffected by the presence of these tumor cells (Figure 3F). These results together confirm an indispensable role for GzB in the tumoricidal function of LF-IC-primed monocytes.

### 2.4. Role of CD32a (FcγRIIa) and Membrane-Bound CD14 in LF-IC Priming of Human Monocytes

**Figure 4:**
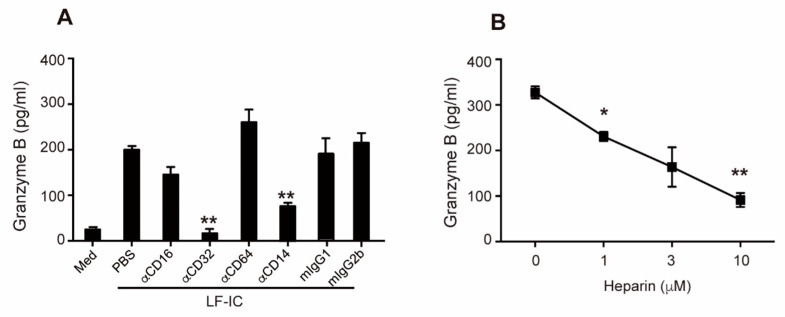
Crosslinking of FcγRIIa and membrane-bound CD14 was essential for the tumoricidal activity empowerment to monocytes by LF-IC. (**A**) Freshly isolated human monocytes were primed with 30 μg/mL LF-IC for 48 h in the presence of 5 μg/mL αCD16, αCD32, αCD64 or αCD14. mIgG1 and mIgG2b were included as isotype controls. (**B**) Monocytes were primed with 30 μg/mL LF-IC for 48 h in the presence of different concentration of heparin. GzB in supernatant was determined by ELISA. * *p* < 0.05, ** *p* < 0.01, *** *p* < 0.001.

Membrane-bound CD14 (mCD14) is a co-receptor of TLR4, they function together as a receptor complex in monocytes for LF [19]. Consistent with our previous finding that LF-IC strongly activates human monocytes through co-ligation of FcγRIIa (but not FcγRI and FcγRIII) and mCD14/TLR4 [19], GzB production by human monocytes under stimulation of LF-IC was significantly inhibited by αCD32a (FcγRIIa), but not αCD64 (FcγRI) or αCD16 (FcγRIII), mAb (Figure 4A). Additionally, mAb against human CD14 almost completely abolished GzB production by LF-IC-primed monocytes (Figure 4A). Meanwhile, heparin, a carbohydrate capable of blocking LF-CD14 interaction, dose-dependently inhibited GzB production by human monocytes under LF-IC stimulation (Figure 4B).

### 2.5. LF-IC Induces GzB Expression in Human Monocytes via the syk-PI3K-AKT Pathway

**Figure 5:**
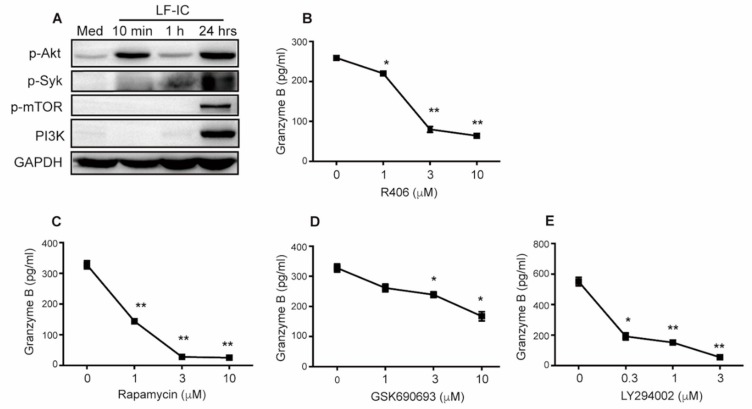
The signaling pathway for the tumoricidal activity empowerment to monocytes by LF-IC. (**A**) Freshly isolated human monocytes were stimulated with 30 mg/mL LF-IC for indicate time. The proteins were extracted and Western blotting analysis of the phosphorylation of syk, PI3K, Akt, and mTOR. (**B**–**E**) Monocytes were primed with 30 μg/mL LF-IC for 48 h in the presence of various concentration of kinase inhibitors, including SYK inhibitor (R406), mTOR inhibitor (rapamycin), AKT inhibitor (GSK690693) and PI3K inhibitor (LY294002). GzB in supernatant was determined by ELISA. * *p* < 0.05, ** *p* < 0.01, *** *p* < 0.001.

Syk is a predominant kinase essential for signal transduction of immunoreceptor tyrosine-based activation motifs (ITAMs) in the cytoplasmic tail of FcγRs [33]. As shown in Figure 5A, LF-IC strongly induced phosphorylation of Akt, Syk and mTOR in human monocytes as evidenced by Western Blotting Assay (Figure 5A). Given also that PI3K was also induced in the same experiment (Figure 5A), it is highly likely that LF-IC elicited GzB production in human monocytes through the PI3K/Akt/mTOR pathway. This is supported by the results that specific inhibitors against Syk (R406), PI3K (LY294002), AKT (GSK690693) and mTOR (rapamycin) significantly decreased GzB expression in LF-IC-primed human monocytes (Figure 5B–E).

### 2.6. Unresponsiveness of Murine Monocytes to LF-IC Stimulation

**Figure 6:**
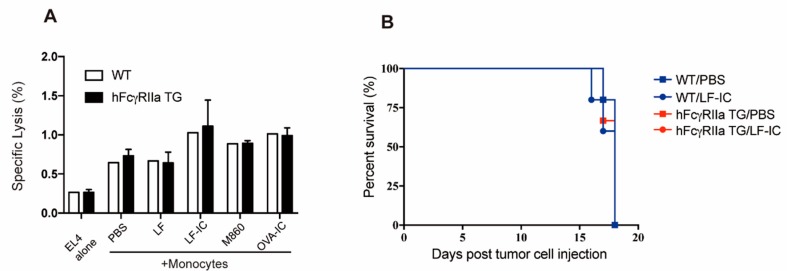
Unresponsiveness of mouse monocytes to LF-IC stimulation. (**A**) Freshly purified mouse monocytes of C57/BL6 wild type (WT) or hFcγRIIa transgenic mice (hFcγRIIa TG) were primed with 30 μg/mL LF, LF-IC, M860, OVA-IC for 24 h, followed by further incubation with EL4 tumor cells for 18 h. Cells were acquired and analyzed by FACS and results were expressed by the percentage of CFSE^+^PI^+^ cell counts in CFSE^+^ cell counts. * *p* < 0.05, ** *p* < 0.01. (**B**) WT or hFcgRIIa TG mice were inoculated with 10^6^ EL4 intravenously. Mice were treated with 100 μg LF-IC (i.p.) at 1 day before inoculation and 1, 3 days post inoculation. Kaplan–Meier survival curves for all 6 mice/groups was shown.

Since mice do not have FcγRIIa homologue, human FcγRIIa transgenic (hFcγRIIa-TG) mice were employed for further investigation on whether LF-IC exhibits anti-tumor effect through augmentation of tumoricidal activity of monocytes/macrophages in vivo. Surprisingly, LF-IC-primed hFcγRIIa TG murine monocytes did not show tumoricidal activity against EL4 cells, a mouse leukemia cell line (Figure 6A). We also compared hFcγRIIa TG mice treated with, or without, LF-IC, for response to intravenous inoculated viable EL4 cells, no significant difference was observed in terms of time of survival (Figure 6B).

## 3. Discussion

Tumorigenesis is a failure of immune surveillance towards malignant cells, therefore improving immune response is expected to have beneficial effect against cancers. LF exhibits functions of immunoregulation and anti-tumor protection [2,3]. However, its therapeutic efficacy is quite low, which has to be compensated by high dosage [8]. In this study, we demonstrate that combination of human LF and M860, a mouse monoclonal IgG1 antibody against human LF, renders human monocytes potent tumoricidal activity through FcγRIIa-CD14/TLR4 co-ligation and induction of GzB. This strategy allows LF to achieve anti-tumor effect at significantly lower concentration. The Fc proportion of mouse IgG1 is able to bind human FcγRIIa [34], which is a low affinity receptor for IgG in ICs. Humanized M860 with increased binding affinity for hFcγRIIa may further enhance the efficiency in facilitating the anti-tumor potential of low dose hLF in vivo.

Mouse monocytes transgenic for hFcγRIIa did not respond to LF-IC in the same way as human monocytes (Figure 6). Our recent data indicate that CD14/TLR4 complex on the surface of murine monocytic cells is also capable of binding hLF (not shown), thereby excluding the possibility that the unresponsiveness of hFcγRIIa-TG mouse monocytes to LF-IC was due to lack of binding with mouse CD14/TLR4. An important consideration here is that FcγRs expressed on the surface of mouse myeloid cells differ considerably from those of human monocytes. For instance, FcγRIIb, an inhibitory FcγR, is expressed at a high level in mouse monocytes, but at a low level in human monocytes [35,36]. This could be a main reason for the poor response of mouse monocytes to LF-IC priming. Humanized mouse models would be needed for future investigation on anti-tumor effects of hLF in combination with M860 in vivo.

Among the cytokines released by LF-IC-primed human monocytes, TNFα could induce cell death of tumor cells. However, TNFα in LF-IC primed monocyte cultures was insufficient to induce cellular death of Jurkat and Raji cells. It has previously been documented that Jurkat cells were insensitive to TNFα or TRAIL [37]. Another possibility is that the amount of TNFα produced by LF-IC-primed monocytes was not large enough to achieve significant tumoricidal effects. Granzymes, a family of serine proteases expressed by cytotoxic cells, play important roles in protection against viral infection and cellular transformation. GzB is the most powerful member of the granzyme family. Our work identifies GzB as the major soluble mediator of the tumoricidal activity of LF-IC-primed monocytes. It has been shown that TLR8 agonist could boost antibody-dependent cell death (ADCC) through the induction of GzB [38]. Our data further emphasize that the interaction of TLRs with FcγRs could be beneficial for conditioning innate immune cells such as monocytes and macrophages for tumoricidal activity. Though cytotoxicity of GzB is not tumor-specific, receptors for LF, such as LRP, are well characterized tumor-associated antigens, which may help effective targeting of the LF-IC-primed monocytes [39,40]. In conclusion, our results on the tumoricidal activity of LF-IC-primed monocytes will expand the clinical cancer therapeutic application of LF and provide new research areas of tumor therapy.

## 4. Materials and Methods

### 4.1. Reagents and Abs

Human LF, LPS, heparin and OVA were taken from Sigma-Aldrich (STL, USA). Mouse IgG1 (ET901), IgG2b (MPC-11), mAbs against human FcγRI (CD64, 10.1) or FcγRIII (CD16, 3G8) were taken from BioLegend (San Diego, CA, USA). Anti-human FcγRIIa mAb (CD32a, IV.3) was taken from STEMCELL Technologies (Vancouver, Canada). Mouse anti-human CD14 mAb (134620) and GzB ELISA kit were taken from R&D (Minneapolis, MN, USA). GzB inhibitor (Z-AAD-CMK) was taken from Merck Millipore (Darmstadt, Germany). SYK inhibitor (R406), mTOR inhibitor (rapamycin), AKT inhibitor (GSK690693) and PI inhibitor (LY294002) were taken from Selleck (Houston, TX, USA). TNFα blocking antibody (Etanercept) was taken from AbMole (Houston, TX, USA).

### 4.2. Peripheral Blood Monocyte Isolation and Cell Culture

Peripheral blood monocytes (PBMs) were obtained from healthy individuals after informed consent in accordance with procedures approved by the human ethics committee of Soochow University. Monocytes were sorted from PBMCs using human αCD14 magnetic-labeled beads (MACS; Miltenyi Biotec) according to the manufacturer’s instructions. The purity of monocytes obtained was around 95%, as determined by flow cytometer with αCD14 mAb. Purified monocytes were cultured in RPMI-1640 medium supplemented with 10% (*v*/*v*) autologous serum, penicillin/streptomycin (100 U/mL), at 37 °C and 5% CO_2_. Human T-cell leukemia Jurkat cells. B-cell leukemia Raji cells and mouse leukemia EL4 cells were originally obtained from the American Type Culture Collection (ATCC). All cells were cultured in RPMI-1640 (Hyclone) supplemented with 10% FBS (BI), penicillin/streptomycin (100 U/mL) at 37 °C with 5% CO_2_.

### 4.3. Mouse Monocyte Isolation

Bone marrow cells from WT and hFcγRIIa TG mice were treated with red blood cell lysis buffer and resuspended in PBS at a density of 10^8^/mL. Monocytes were enriched using EasySep^TM^ Mouse Monocyte Isolation Kit (StemCell Technology, Vancouver, Canada). In brief, cell suspension incubated with selection cocktail including biotin labeled anti-CD3e, CD45R, Ly-6G, NK1.1, CD117 and Siglec F, followed by incubation with streptavidin coupled beads. The monocytes were purified using magnet depleting other cell population in bone marrow.

### 4.4. Anti-Tumor Effects

Freshly purified human monocytes or mouse monocytes were seeded at a density of 10^5^ cells per well in a 96 well plate and primed with stimulators for 24 h. 2 × 10^4^ CFSE labeled Jurkat or Raji were later added and co-cultured with primed human monocytes for 18 h. 2 × 10^4^ CFSE labeled EL4 cells were added and co-cultured with freshly purified mouse monocytes for 18 h. Cells were acquired and analyzed by FACS with PI (Propidium Iodide) staining. Results were expressed by the percentage of CFSE^+^PI^+^ cell counts in CFSE^+^ cell counts.

### 4.5. ELISA

The concentration of human Granzyme B in the culture supernatant was determined using ELISA kits (Minneapolis, R&D, USA). Cell supernatants were collected and centrifuged at 10,000 g to clear cellular debris. ELISAs were performed according to the respective manufacturer protocols.

### 4.6. Real-Time RT-PCR

RNA was isolated from cells with the Total RNA Kit II (OMEGA, Norcross, GA, USA) and subsequently reversely transcribed into cDNA using an oligo (dT) primer (Takara, JP). For mRNA-level analysis, cells were lysed at the indicated time points, after which mRNA extraction was performed. Q-PCR (Life Technology, USA) was performed using SYRB green (Takara, JP) and primer pairs as listed in Table 1.

### 4.7. Tumor Models

WT or hFcγRIIa TG mice were obtained from Jackson Laboratory (Bar Harbor, ME, USA) and bred with C57/BL6 mice for more than 10 generations. WT and hFcγRIIa TG (n = 12) were inoculated with 10^6^ EL4 intravenously. Mice were treated with 100 μg LF-IC or PBS i.p. at the day before inoculation and 1, 3 days post inoculation. Survival of the animals were monitored for 20 days post inoculation.

### 4.8. Western Blotting Analysis

Cells were lysed in lysis buffer. After centrifugation at 12,000 g for 15 min at 4 °C, the supernatant was collected, and its protein concentration was determined using BCA Protein Assay Kit (Pierce, Rockford, IL, USA). 30 μg proteins for each sample, were separated on 10% SDS-PAGE and transferred onto polyvinylidene difluorid (PVDF) membranes (Millipore, Bedford, MA, USA), followed by being blocked with 5% bovine serum albumin in PBST (0.1% Tween-20 in PBS) for 1 h. The membranes were incubated with anti-p-Syk, p-PI3K, p-Akt, p-mTOR and β-actin (Cell signaling technology, Danvers, MA, USA) at 4 °C overnight followed by incubation with horseradish peroxidase-conjugated secondary antibody for 1 h at room temperature. The blots were developed by enhance chemiluminscence detection regents (Pierce, Rockford, IL, USA).

### 4.9. Study Approval

This study was approved by the Ethics Committees of Soochow University, Suzhou, China. The methods were carried out in accordance with the guidelines of Soochow University. Written informed consent was obtained from all participants prior to inclusion in the study.

### 4.10. Statistical Analysis

Data were expressed as the mean ± SEM of at least three independent experiments. The student’s *t*-test was used to compare differences among groups by using GraphPad Prism 5.0 software and values at *p* < 0.05 were considered significant.

## 5. Conclusions

Data presented here demonstrate that IC between hLF and M860, an anti-hLF mAb, renders human monocytes tumoricidal activiy via induction of secreted GzB. LF-IC is able to colligate CD14/TLR4 and FcγRIIa on the surface of human monocytes, thereby triggering the Syk-PI3K-AKT-mTOR pathway leading to GzB production. Our results may help to expand cancer therapeutic application of low dosage LF.

## Figures and Tables

**Table 1:** Primers for indicated genes.

	Forward (5′-3′)	Reverse (5′-3′)
hGAPDH	GAGTCAACGGATTTGGTCGT	TTGATTTTGGAGGGATCTCG
hGzA	AACCAGGAACCATGTGCCAA	GGCTTCCAGAATCTCCATTGC
hGzB	GAGCAAGGAGGAAACAACAGC	GGCCCCCAAGGTGACATTTA
hGzH	ATGCTACTGCAGGGGGACT	TCAGGCCCAGAGGAAGGTTA
hGzK	CCCTGCGAGAAGTCACTGTT	CCCCCTGAGTCACCCTTACA
hGzM	GTCAGTAGCTCCTTTGGGACC	GGCTGTTGTTACACATGCGG
